# Editorial: Radioembolization in the Treatment of Liver Cancer: A Multidisciplinary Approach for Individualized Therapy

**DOI:** 10.3389/fonc.2015.00216

**Published:** 2015-10-08

**Authors:** Cicero M. R. Habito

**Affiliations:** ^1^Department of Radiology, Harvard Medical School, Massachusetts General Hospital, Boston, MA, USA

**Keywords:** radioembolization, SIRT, Y90, PET, personalized medicine, precision medicine

Radioembolization has certainly come a long way since its early years of development in Australia and Asia, where the incidence and mortality rates of hepatic malignancies remain among the highest globally (1). Now, a necessary arsenal component of cancer centers worldwide, it has evolved from its original indication for liver tumors, and has shown utility for tumors in other organs as well.

In the purest sense, radioembolization has enabled interventional radiologists to use beta emissions to radiate malignancies refractory to conventional treatments. It has, however, also led to the challenging dilemma of figuring out how to make sure lesions are adequately radiated while maintaining the requisite safety profile. Achieving the utopian combination of highest possible dose to tumor and lowest possible dose to unaffected tissue has been more complex than initially thought. We now know that standard scintigraphic imaging is unable to provide reliable quantitative dosimetry estimates. Furthermore, we have learned through years of practice that tissue and tumor radiation thresholds vary with certain clinical and histologic parameters. Coupled with recent advances in positron emission tomography (PET) imaging, these findings have brought radioembolization right to the doorstep of personalized medicine.

This research topic puts together a collection of papers which, when taken together, bring a personalized approach to radioembolization within arm’s reach of multidisciplinary treatment centers. The author list is illustrative of the synergy among experts in different fields, which is required for treatment to be successful – including Oncology, Diagnostic and Interventional Radiology, Nuclear Medicine and Medical Physics. As an editor, I am particularly proud to note the fact that this project represents a truly international effort, with authors and reviewers representing centers which have been blazing trails in radioembolization in Asia, Europe, Australia, and the Americas.

Two articles lay the groundwork with an overview of radioembolization as applied to malignancies. One is dedicated to hepatic metastases from colorectal primary malignancies (2) and the other looks at publications on radioembolization of both extrahepatic tumors and hepatic tumors of non-colorectal or hepatocellular primary origin (3).

A detailed review of data and outcomes that state the case for radioembolization’s role beyond palliation, as a tool enabling definitive hepatic resection or successful transplantation, is provided (4).

Marta Cremonesi and her Italy-based medical physicist colleagues have contributed a critical review of radioembolization from a radiobiologic and dosimetric perspective (5). Significantly, the authors discuss the scientific basis for a paradigm shift to individualized approaches to dosimetry and risk stratification. This is ideally read in tandem with a review article on the side effects of ^90^Y radioembolization (6), and followed by an article describing the use of hepatobiliary scintigraphy as a tool for imaging regional liver function following radioembolization (7).

The rapid growth of PET/CT as a mainstream imaging modality has positioned it well for a routine role in post-radioembolization imaging, with many suggesting it should replace scintigraphy as the next gold standard. This has made radioembolization a wide open field for PET research. It is, therefore, no coincidence that four articles in this collection are rooted in utilization of PET/CT imaging in conjunction with radioembolization.

Pasciak et al. have provided a must read article on the role of PET/CT in tandem with radioembolization, beginning with a concise review of the physics of ^90^Y positron emission, followed by a review of the clinical utility of the modality and the promise it holds for improving radioembolization outcomes (8). Anyone engaged in research in the field will find a valuable signpost in the author’s suggestions for improving ^90^Y PET/CT imaging.

Those interested in seeing PET/CT at work following radioembolization are certain to appreciate images from actual cases. These are included together with a description of an image assessment technique for detection of non-target deposition of ^90^Y microspheres using PET/CT following radioembolization (9).

Two prospective research papers are included, which are sure to be references for future PET/CT dosimetry initiatives: one compares techniques for ^90^Y PET/CT Image-Based Dosimetry Following Radioembolization (10), and the other describes a method for determination of radiation dose to both tumor and unaffected liver tissue using PET following ^90^Y radioembolization (11).

Completing the loop, lest we fail to appreciate the value that scintigraphy brings to radioembolization, is a contribution by Walrand et al. Their article provides an early indication of how simulations can advance planar imaging, and keep hopes alive for the ability to perform dosimetry assessments without needing to move patients off the catheterization table (12).

If this research topic is any indication, the once seemingly cookie cutter approach to radioembolization is now a thing of the past. Instead, radioembolization will soon be to me as a suit is to a tailor and a meal is to a chef: uniquely adapted to the patient, every step of the way.

## Conflict of Interest Statement

The author declares that the research was conducted in the absence of any commercial or financial relationships that could be construed as a potential conflict of interest.
